# Identification and validation of genetic signature associated with aging in chronic obstructive pulmonary disease

**DOI:** 10.18632/aging.204358

**Published:** 2022-10-28

**Authors:** Shanshan Chen, Yuan Zhan, Jinkun Chen, Jixing Wu, Yiya Gu, Qian Huang, Zhesong Deng, Xiaojie Wu, Yongman Lv, Jungang Xie

**Affiliations:** 1 Department of Respiratory and Critical Care Medicine, National Clinical Research Center of Respiratory Disease, Key Laboratory of Pulmonary Diseases of Health Ministry, Tongji Hospital, Tongji Medical College, Huazhong University of Science and Technology, Wuhan 430030, Hubei, China; 2 Department of Science, Western University, London, Ontario N6A 3K7, Canada; 3 Department of Respiratory and Critical Care Medicine, Wuhan No.1 Hospital, Wuhan Hospital of Traditional Chinese and Western Medicine, Wuhan 430022, China; 4 Health Management Center, Tongji Hospital, Tongji Medical College, Huazhong University of Science and Technology, Wuhan 430030, Hubei, China

**Keywords:** chronic obstructive pulmonary disease, aging, bioinformatics analysis, genetic signature

## Abstract

Aging plays an essential role in the development for chronic obstructive pulmonary disease (COPD). The aim of this study was to identify and validate the potential aging-related genes of COPD through bioinformatics analysis and experimental validation. Firstly, we compared the gene expression profiles of aged and young COPD patients using two datasets (GSE76925 and GSE47460) from Gene Expression Omnibus (GEO), and identified 244 aging-related different expressed genes (DEGs), with 132 up-regulated and 112 down-regulated. Then, by analyzing the data for cigarette smoke-induced COPD mouse model (GSE125521), a total of 783 DEGs were identified between aged and young COPD mice, with 402 genes increased and 381 genes decreased. Additionally, functional enrichment analysis revealed that these DEGs were actively involved in COPD-related biological processes and function pathways. Meanwhile, six genes were identified as the core aging-related genes in COPD after combining the human DEGs and mouse DEGs. Eventually, five out of six core genes were validated to be up-regulated in the lung tissues collected from aged COPD patients than young COPD patients, namely NKG7, CKLF, LRP4, GDPD3 and CXCL9. Thereinto, the expressions of NKG7 and CKLF were negatively associated with lung function. These results may expand the understanding for aging in COPD.

## INTRODUCTION

Chronic obstructive pulmonary disease (COPD) is a progressive and debilitating respiratory disease that causes a heavy burden both medically and economically [1]. By 2050, the life expectancy of the global population will be extended, and about 15% of the world’s population will reach 70 years or more [2]. According to the Global Burden of Disease Study (the GBD study), the incidence and mortality of COPD patients over 70 years old are significantly higher than those of young patients. It can be discovered that aging increases the clinical incidence and treatment difficulty of COPD [3–6]. Therefore, a better understanding of the pathophysiology of COPD and knowing how the aging process is related to the COPD development are beneficial and necessary for the clinical treatment of this disease.

Although the association between aging and COPD has been repeatedly proposed, the mechanism of this association remains unclear [5, 6]. Lopez-Otin et al. summarized 9 manifestations associated with aging, including genomic instability, telomere loss, epigenetic changes, loss of protein homeostasis, demodulating nutritional sensing, mitochondrial dysfunction, cell senescence, stem cell failure and changes in intercellular communication [7]. Studies have shown that the telomere length of patients with COPD is shorter than that of healthy people [8, 9]. In addition, cigarette smoke has been shown to induce the expression of aging markers in lung epithelial cells and fibroblasts, including senescence-related secretory phenotypes, p21 and p16 [10, 11]. However, although it is generally believed that there is a link between aging and COPD, the underlying mechanism is not clear [12–14].

In the current study, in order to explore the underlying mechanism between aging and COPD development, using gene expression profiles of COPD patients and COPD model mice, we analyzed the genetic signature related to aging and corresponding enrichment biological processes, as well as the validations in the clinical specimens.

## RESULTS

### DEGs associated with aging in COPD patients

**Figure 1:**
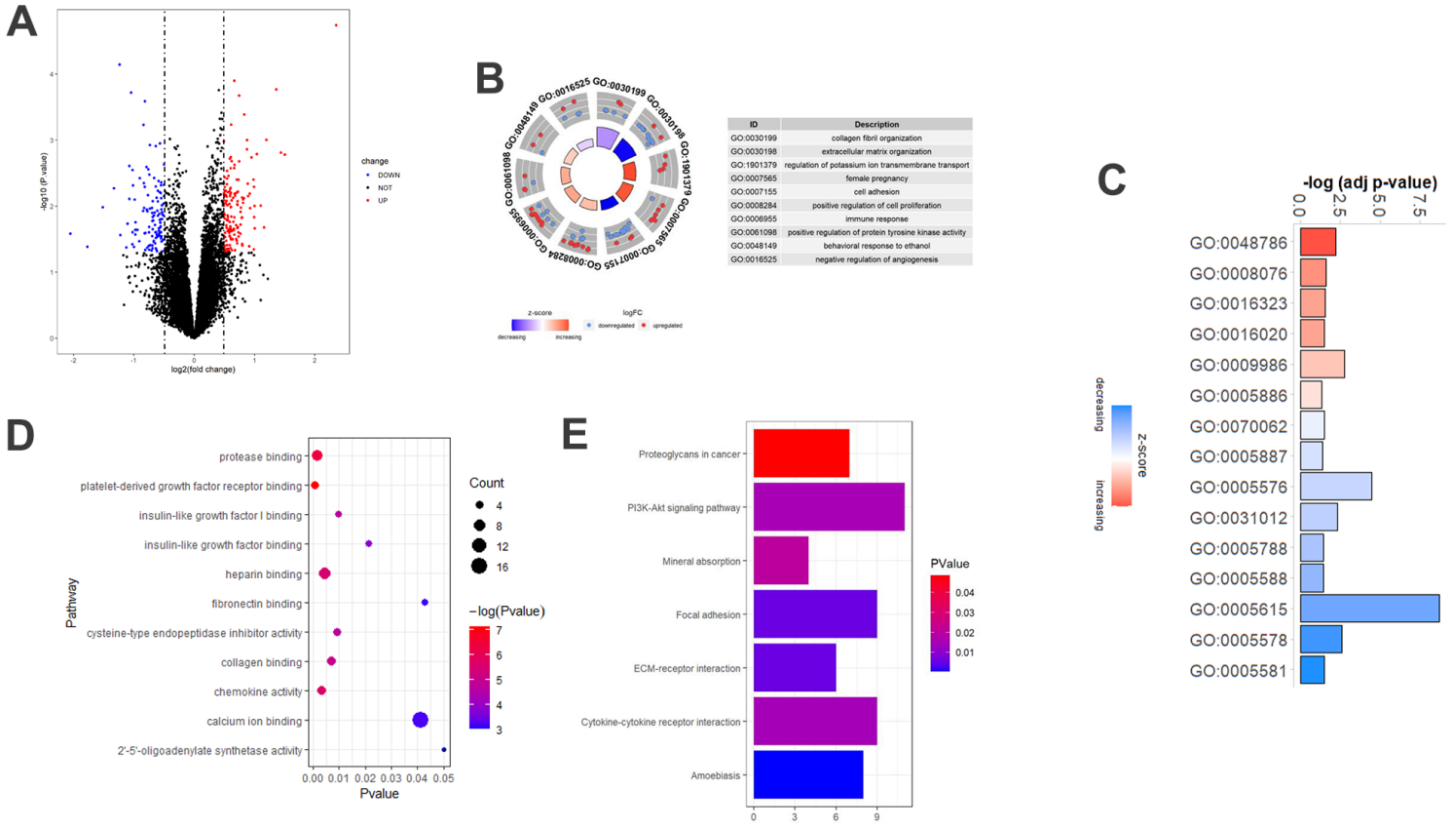
**DEGs associated with aging in COPD patients.** (**A**) Volcano-plot of the 244 age-associated DEGs in COPD patients. Red circle: upregulated genes; blue circles: downregulated genes. (**B**) Top 10 GO biological processes analysis of the 244 age-associated DEGs. Outer gray circle: a scatter plot for each term of the logFC of the assigned genes; red circles: upregulated genes; blue circles: downregulated genes. (**C**) All GO cellular components analysis of the 244 age-associated DEGs. (**D**) Top 11 GO molecular functions analysis of the 244 age-associated DEGs. (**E**) Top 7 significantly (P<0.05) enriched KEGG pathways. DEGs, differently expressed genes; COPD, chronic obstructive pulmonary disease; GO, gene ontology; FC, fold change; KEGG, Kyoto Encyclopedia of Genes and Genomes.

To determine the aging-related core gene profiles of COPD patients, two microarray datasets were obtained from GEO. The information included in the study was listed in Table 1. According to age criteria, this study included 11 young patients with COPD and 66 aged patients with COPD, and the detailed information regarding the included subjects can be accessed in Supplementary File 1. Comparing the gene expression profiles of the two groups, 244 DEGs were generated, of which 132 were up-regulated and 112 were down-regulated (Figure 1A). In order to further understand the functions related to the 244 DEGs, GO enrichment analysis was carried out, including BP, MF and CC. In BP analysis, the DEGs were found to associate with “collagen fibril organization”, “extracellular matrix organization”, and “regulation of potassium ion transmembrane transport” (Figure 1B). In the CC category, the DEGs were related to the “extracellular space”, “extracellular region”, and “cell surface” (Figure 1C). Moreover, DEGs were enriched in the MF category related to “calcium ion binding”, “heparin binding”, and “protease binding” (Figure 1D). For KEGG pathway enrichment analysis, the top three significant KEGG pathways of the DEGs included “PI3K-AKT signaling pathway”, “Cytokine-cytokine receptor interaction”, and “Focal adhesion” (Figure 1E).

**Table 1:** Characteristics of microarray datasets included.

**Dataset**	**Platform**	**Samples**	**Sample number (Young group)**	**Sample number (Aged group)**
GSE76925	GPL10558	Lung tissue of COPD patient	2	12
GSE47460	GPL14550	Lung tissue of COPD patient	9	54
GSE125521	GPL6885	Lung tissue of CS-exposed mouse	3	3

COPD, chronic obstructive pulmonary disease; CS, cigarette smoke.

### DEGs associated with aging in COPD model mice

**Figure 2:**
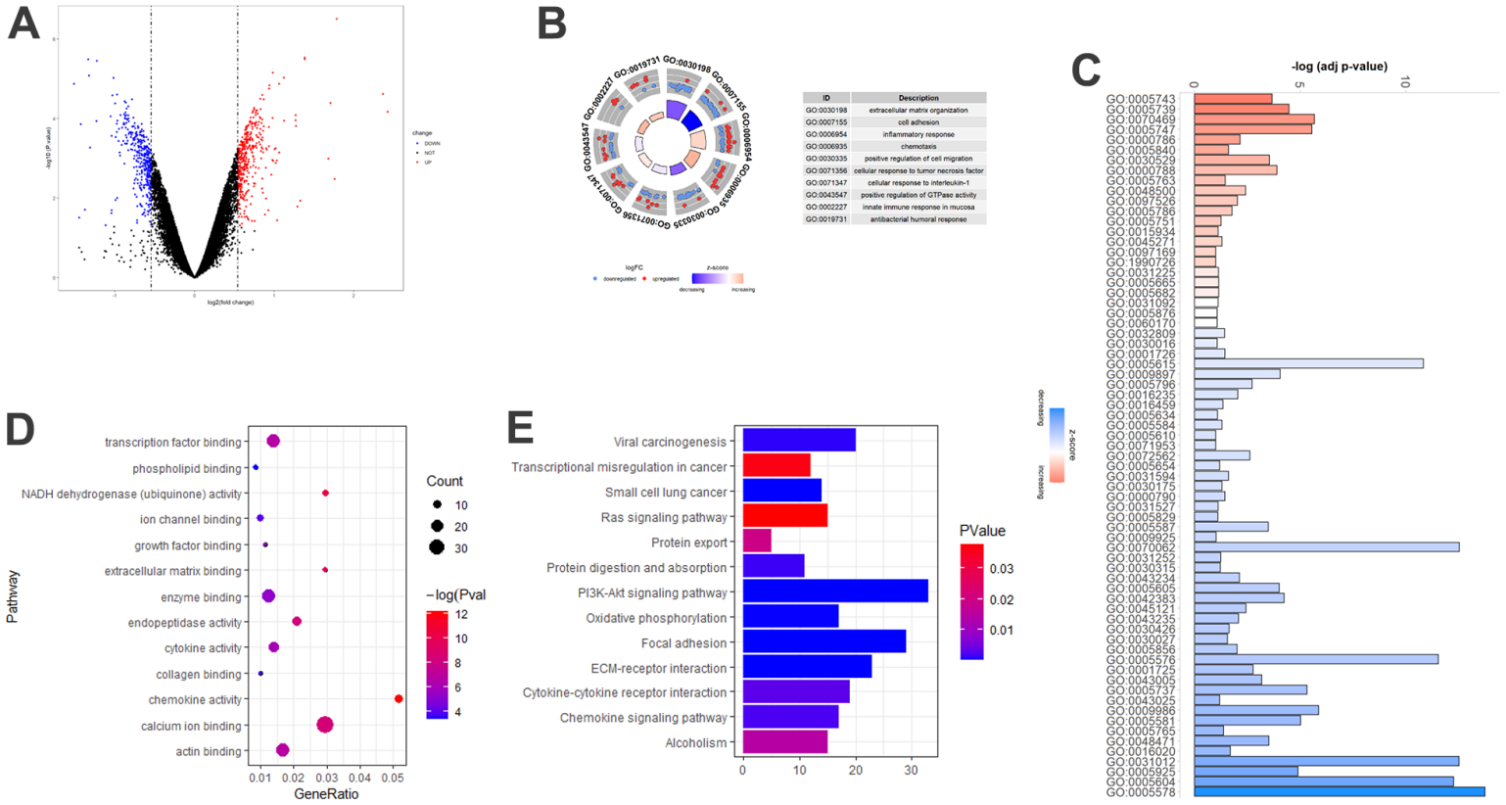
**Age-associated DEGs in COPD model mice.** (**A**) Volcano plot of the 783 age-associated DEGs. Red circle: upregulated genes; blue circles: downregulated genes. (**B**) Top 10 GO biological processes of the 783 age-associated DEGs. Outer gray circle: a scatter plot for each term of the logFC of the assigned genes; red circles: upregulated genes; blue circles: downregulated genes. (**C**) All GO cellular components of the 783 age-associated DEGs. (**D**) Top 13 GO molecular functions of the 783 age-associated DEGs. (**E**) Top 13 significantly (P<0.05) enriched KEGG pathways. DEGs, differently expressed genes; COPD, chronic obstructive pulmonary disease; GO, gene ontology; FC, fold change; KEGG, Kyoto Encyclopedia of Genes and Genomes.

To further identify the expression characteristics related to aging in COPD model mice, we obtained a dataset from GEO and compared the DEGs between aged and young COPD mice, and the detailed information regarding the included subjects can be accessed in Supplementary File 2. A total of 783 DEGs were identified, of which 402 genes increased and 381 genes decreased (Figure 2A). Slightly different from the results obtained from patients with COPD, the top three BP terms were enriched in “extracellular matrix organization”, “cell adhesion”, and “inflammatory response” (Figure 2B). In the CC category, the DEGs were associated with “proteinaceous extracellular matrix”, “extracellular exosome”, “extracellular matrix”, “basement membrane”, and “extracellular region” (Figure 2C). “Calcium ion binding”, “actin binding”, “enzyme binding”, and “transcription factor binding” were the most important MF terms (Figure 2D). Furthermore, KEGG pathway analysis showed enrichments in “PI3K-Akt signaling pathway”, “Focal adhesion”, and “ECM-receptor interaction” (Figure 2E).

### Core DEGs associated with aging in COPD

**Figure 3:**
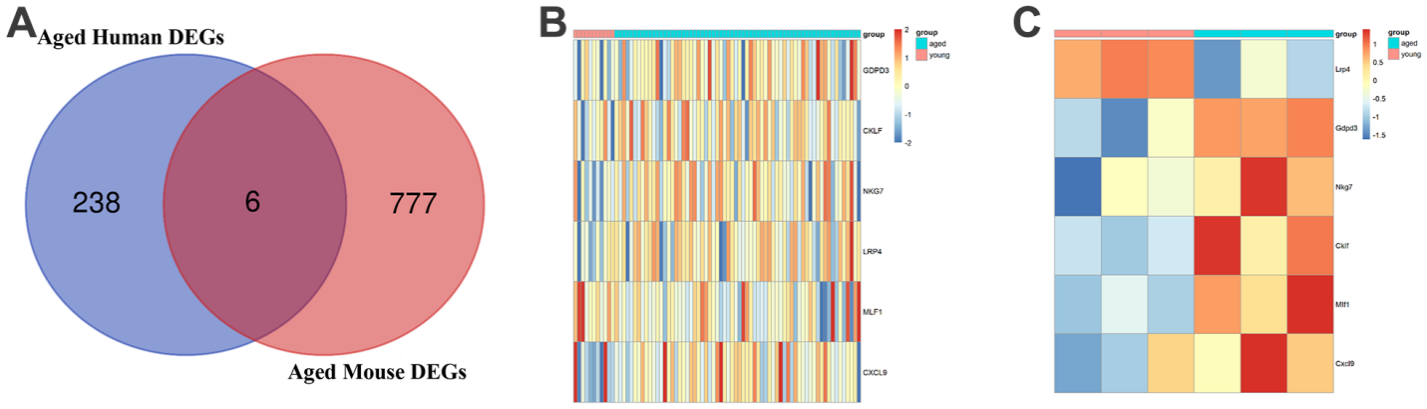
**Common age-associated DEGs in COPD.** (**A**) A Venn diagram of DEGs of patients with COPD patients and COPD model mice. (**B**) Heatmap of the six core genes in young and aged COPD patients. (**C**) Heatmap of the six core genes in young and aged COPD model mice. DEGs, differently expressed genes; COPD, chronic obstructive pulmonary disease.

To comprehensively explore the gene expression profile in COPD related to aging, we combined the results of COPD patients and COPD model mice. As shown in the Venn diagram, six common DEGs were observed both in aged COPD patients and aged COPD mice (Figure 3A). The heat maps showed the gene expression profile of six aging-related genes in COPD patients and COPD mice (Figure 3B, 3C). Details of the six core genes were shown in Table 2.

**Table 2:** Detailed information of 6 core genes.

**Human**						**Mouse**			
**Gene**	**Full name**	**ID**	**Log_2_FC**	**P value**		**Gene**	**ID**	**Log_2_FC**	**P value**
NKG7	natural killer cell granule protein 7	4818	0.71906	0.00841		Nkg7	72310	0.55968	0.01462
CKLF	chemokine like factor	51192	0.55955	0.01459		Cklf	245978	0.67981	0.00104
LRP4	LDL receptor related protein 4	4038	0.65921	0.02236		Lrp4	228357	-0.62272	0.00088
MLF1	myeloid leukemia factor 1	4291	-0.61519	0.04810		Mlf1	17349	0.72495	0.00051
GDPD3	Glycerophosphodiester phosphodiesterase domain containing 3	79153	0.53665	0.01607		Gdpd3	68616	0.63428	0.00119
CXCL9	C-X-C motif chemokine ligand 9	4283	1.15805	0.02108		Cxcl9	17329	0.66788	0.03170

Human, Aged COPD patient vs Young COPD patient; Mouse, Aged CS-expose mouse vs Young CS-expose mouse; FC, fold change.

### Subject characteristics

The clinical characteristics of all subjects were displayed in Table 3. In all, 32 subjects were included in the current study containing 16 young COPD patients and 16 aged COPD patients. The subjects presented no obvious differences by gender, body mass index (BMI) and smoke status. The patients in aged COPD group had significantly lower FEV_1_% of predicted and FEV_1_/FVC, compared with those in the young COPD group. Meanwhile, the aged COPD group had a greater proportion of patients with higher GOLD grade (including grade II and III), relative to the young COPD group. Collectively, the aged COPD patients had worse lung function and more severe disease progression.

**Table 3:** Clinical characteristics of subjects.

	**Young COPD (n=16)**	**Aged COPD (n=16)**	**P value**
Age (years)	47.88 (3.05)	72.31 (2.33)	<0.0001
Gender (male/female)	9/7	11/5	0.72
BMI (kg/m^2^)	23.16 (2.05)	22.99 (2.50)	0.84
Non-smokers/smokers	8/8	5/11	0.47
FEV1 (% predicted)	94.58 (19.83)	70.83 (19.63)	0.0019
FEV1/FVC (%)	66.79 (2.67)	58.94 (8.11)	0.0009
GOLD Grading			
I	12	5	
II	4	8	
III	0	3	
IV	0	0	

Data are expressed as mean (SD). P-values were calculated using chi-square test or Student’s t-test. COPD, chronic obstructive pulmonary disease; BMI, body mass index; FEV1, forced expiratory volume in one second; FVC, forced vital capacity.

### Validations of the aging-related DEGs

**Figure 4:**
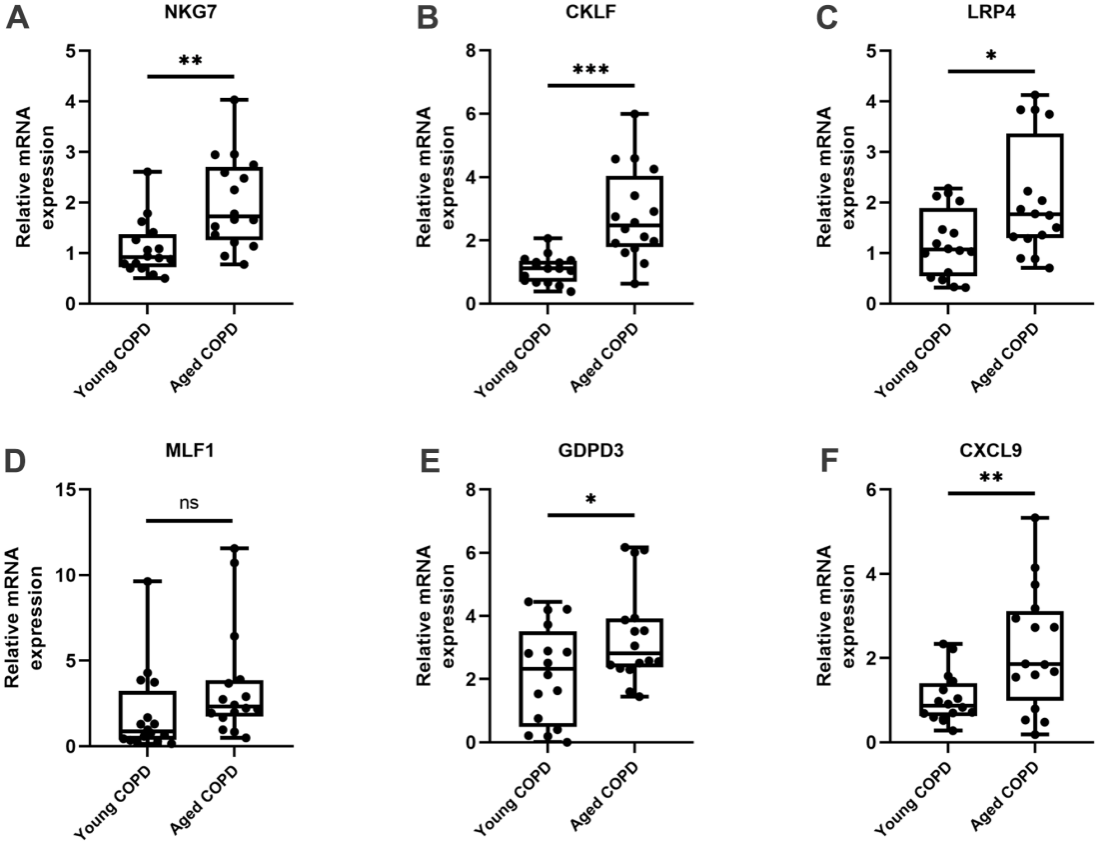
**The expression of the 6 core genes in the lungs of COPD patients.** The relative mRNA expressions of six genes in the young COPD patients and aged COPD patients were shown, including (**A**) NKG7, (**B**) CKLF, (**C**) LRP4, (**D**) MLF1, (**E**) GOPD3, (**F**) CXCL9. Results were expressed as median (P25 quartile, P75 quartile); n=16 for young COPD and n=16 for aged COPD; *P<0.05, **P<0.01, ***P<0.001. COPD, chronic obstructive pulmonary disease; NKG7, natural killer cell granule protein 7; CKLF, chemokine like factor; LRP4, LDL receptor related protein 4; MLF1, myeloid leukemia factor 1; GDPD3, glycerophosphodiester phosphodiesterase domain containing 3; CXCL9, C-X-C motif chemokine ligand 9.

**Figure 5:**
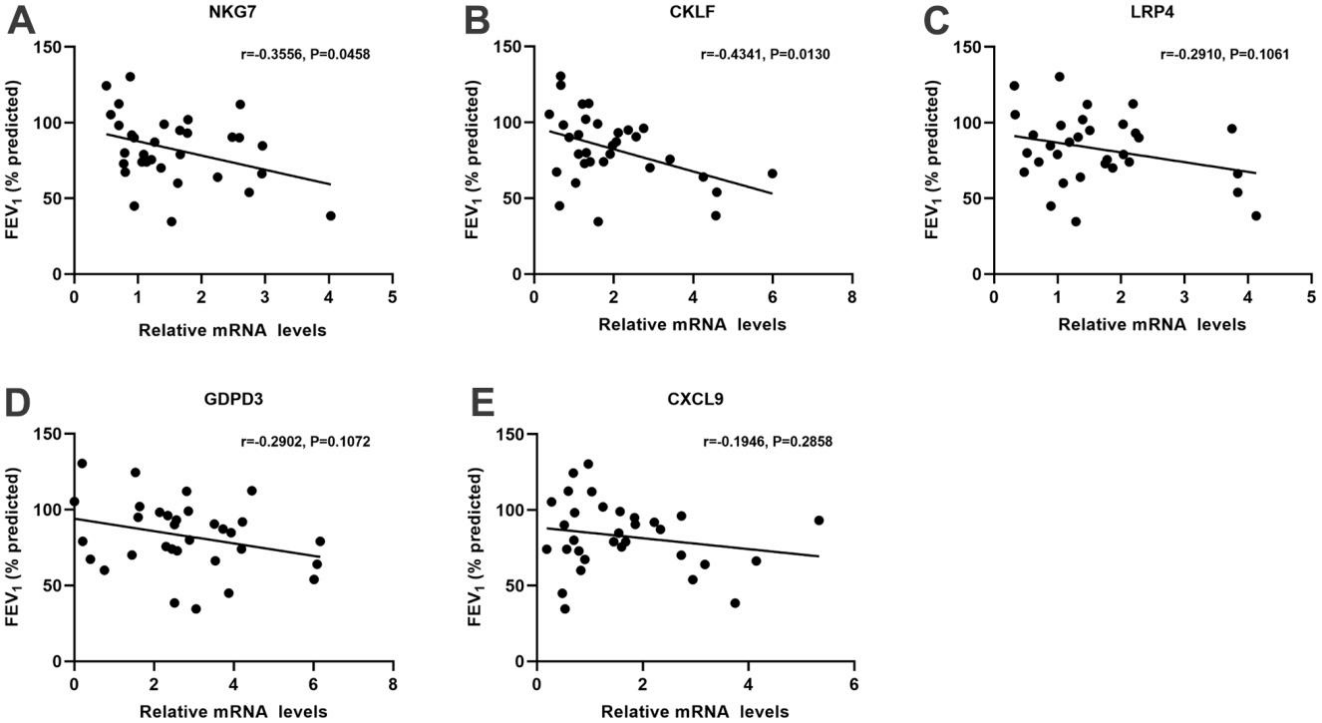
**The association analysis between the critical aging-related DEGs and lung function in COPD patients.** The lung function was negatively associated with (**A**) NKG7 expression and (**B**) CKLF expression, whereas no significant associations with the expressions of (**C**) LRP4, (**D**) GOPD3 and (**E**) CXCL9. DEGs, differently expressed genes; COPD, chronic obstructive pulmonary disease; FEV_1_ (% predicted), forced expiratory volume in 1 s (FEV1) % predicted; NKG7, natural killer cell granule protein 7; CKLF, chemokine like factor; LRP4, LDL receptor related protein 4; GDPD3, glycerophosphodiester phosphodiesterase domain containing 3; CXCL9, C-X-C motif chemokine ligand 9.

The expressions of the 6 aging-related DEGs (NKG7, CKLF, LRP4, MLF1, GDPD3 and CXCL9) were validated in the lung tissues from aged COPD patients and young COPD patients by qRT-PCR. Compared with young COPD patients, the expressions of NKG7, CKLF, LRP4, GDPD3 and CXCL9 were significantly enhanced in aged COPD patients, whereas there’s no marked difference in terms of MLF1 expression (Figure 4). Thereafter, the correlation analysis between lung function and the expression of confirmed core genes was performed. Thereinto, NKG7 and CKLF were negatively correlated with lung function (Figure 5).

## DISCUSSION

Aging is a strong risk factor and independent prognostic biomarker for progressive COPD [15]. However, there is a lack of comprehensive analysis based on gene expression profiles to investigate the role of aging in COPD. In this study, using two human COPD datasets, we identified 244 aging-related DEGs in COPD patients. Similarly, through the analysis of the dataset of COPD model mice, we identified 783 DEGs related to aging. Subsequently, GO and KEGG analyses were conducted regarding these DEGs respectively. Finally, through the combination of DEGs between human and mouse datasets, we identified six common aging-related genes in COPD, of which five genes were confirmed to be up-regulated in clinical specimens, and the expressions of two genes were negatively correlated with lung function in COPD patients.

In this study, 244 aging-related DEGs in COPD patients were involved in the regulation of potassium channel transport in BP function. Previous studies have also shown that potassium exchange was slower in patients with chronic obstructive pulmonary disease than in healthy individuals, taking at least 48 hours [16]. Qiunan Zuo et al. also found that COPD was related to “collagen fibril organization” and “extracellular matrix organization” [17]. Presynaptic components involved most of the top five enriched CC terms, indicating that the nervous system could play an important role in aged patients with COPD. Animal studies have shown that α 2-adrenoceptor agonists may help reduce airway responsiveness in chronic obstructive pulmonary disease, especially when nerve-mediated airway reflexes may be triggered [18]. In addition, a randomized controlled trial found that lumbar percutaneous nerve stimulation improved motor performance in patients with COPD [19]. Platelet growth factor is a significantly enriched MF term. Genes related to platelet growth factor activity were observed in elderly-related diseases [20]. KEGG analysis showed that the DEGs were enriched in PI3K-AKT signaling pathway. Some studies have shown that PTEN/PI3K/AKT can regulate the polarization of macrophages in emphysema mice [21] and induce apoptosis of alveolar epithelial cells in COPD by regulating autophagy through PI3K/AKT/mTOR pathway [22]. Extracellular matrix promotes the proliferation, migration and adhesion of airway smooth muscle cells in rat model of chronic obstructive pulmonary disease through up-regulation of PI3K/AKT signal pathway [22].

Different from the DEGs from COPD patients, the GO enrichment of DEGs in COPD model mice was significant in extracellular matrix (ECM) organization and inflammatory response. Sustained ECM accumulation was thought to be closely involved in the development of several diseases, including COPD, making it difficult to reverse the disease progression of these diseases [23, 24]. Although the relationship between ECM disorders and the aging process is widely recognized, the underlying mechanism remains unclear. The extensive induction of inflammation was revealed to be associated with the remodeling of the epigenome and transcriptome landscape of mouse aging [25]. Age-related chronic inflammation is the main cause of different chronic diseases with the increase of age [26, 27]. There is extensive evidence that persistent inflammation exists in the elderly and that age-related inflammation can occur in the lungs [28].

In our study, we identified six critical aging-related genes in COPD through the bioinformatic analysis with combination of human DEGs and mouse DEGs, and five out of these six genes were validated to be up-regulated in the lung tissues of aged COPD patients, of which NKG7 and CKLF presented remarkably negative associations with lung function. Meanwhile, these coincident genes were more or less related to inflammatory response. In specific, NKG7 was reported to be expressed in various immune cells including activated T cells and NK cells [29], and played important roles in multiple immune-related diseases, like controlling intratumor T-cell accumulation and activation, the cytotoxicity of lymphocytes and regulation of inflammation [30–32]. Future researches are required to validate the possible regulation of NKG7 in airway inflammation, the typical phenotype in COPD pathogenesis. Moreover, CKLF was a novel cytokine which had a crucial role in immune and inflammatory responses [33]. The enhanced CKLF expression was observed to associate with excess production of numerous inflammatory cytokines and graver autoimmune injury in rheumatoid arthritis and asthma [34, 35]. And meanwhile CKLF was also reported to be involved in chemokine activity and neutrophil chemotaxis [36, 37]. In addition, it has been reported that CXCL9 is also associated with chemokine activity and neutrophil chemotaxis [38, 39]. Many studies have proved that neutrophil chemotaxis is simultaneously one of the most important hallmarks and closely related to COPD development [40, 41]. Moreover, CXCL9 was an inflammatory cytokine that played an important role in multiple inflammatory diseases. CXCL9 has been reported to be upregulated in inflammatory colitis [42], and the increased CXCL9 expression was obviously associated with the survival and prognosis of patients with idiopathic pulmonary arterial hypertension (PAH) and chronic thromboembolic pulmonary arterial hypertension (CTEPAH) [43]. Besides, LRP4 and GDPD3 were reported to exert important regulations in lung development. LRP4 is a regulator of Wnt signaling, regulating the differentiation of goblet cells during the lung development and repairment [44, 45]. And meanwhile, goblet cell metaplasia and mucus hypersecretion are the critical features of respiratory diseases, including COPD [46, 47]. In addition, tobacco smoke exposure can promote the disease progress by affecting the expression of Huntingtin-Interacting Protein 1 (HIP1), which could influence GDPD3 expression [48, 49]. Therefore, these 5 aging-related genes are more or less associated with COPD development. But the specific roles of these genes in COPD merit more investigations, which may provide new insights for seeking the therapeutic targets for aged COPD patients.

There are still some limitations in this study. First, we cannot evaluate the role of other aging markers in COPD, such as telomere loss, epigenetic changes and genomic instability, which can be more beneficial to understanding the roles of aging in COPD development. While we only explored the role of aging in COPD from the perspective of related gene transcription. Secondly, the number of clinical samples included in our study is limited, and we need to confirm our conclusions in a larger COPD cohort. Third, we only verified the expression level of the differentially expressed aging-related genes in clinical samples, but did not explore the potential mechanism of these genes in COPD by *in vitro* cell experiments and *in vivo* mouse models. Therefore, on the basis of the above studies, considering the key role of these six aging-related genes, further research may focus on exploring the corresponding mechanisms underlying the association between aging and COPD.

In summary, it is generally believed that aging is a dependent risk factor for COPD. From the point of view of gene expression profile, the pathogenesis of COPD in young patients and aged patients is not exactly the same. Through the combination of bioinformatic analysis, clinical experiment validations and correlation analysis with lung function, our results may provide new potential therapeutic targets for COPD patients with old age.

## MATERIALS AND METHODS

### Microarray data acquisition and process

To identify the genes associated with aging in COPD patients, two microarray datasets (GSE76925 and GSE47460) were downloaded from Gene Expression Omnibus (GEO, http://www.ncbi.nlm.nih.gov/geo) [50, 51]. Young COPD patients were defined as ≤ 50 years old, and aged COPD patients were defined as ≥ 70 years old. To explore age-related genes in COPD model mice, we downloaded a microarray dataset (GSE125521) from GEO. In this dataset, the mice were divided into young group (4-month-old) and aged group (15-month-old) respectively. The details of the GSE datasets were summarized in Table 1. The preprocessing expression matrix and detector annotation files of three GSE datasets were obtained from the GEO repository. The official genetic symbols were used to list probes from different datasets. The multiple expression results of the gene were replaced by the median of the expression results. All the log2 folding changes (log_2_FC) of the expressed results were normalized using limma R packages [52]. Different expression genes (DEGs) were screened according to the cut-off values of p<0.05 and |log_2_FC|>0.5. Gene ontology (GO) function analysis and Kyoto encyclopedia of genes and genomes (KEGG) analysis were also. In order to identify the DEGs between the young and aged groups, the limma R packages (http://www.bioconductor.org/packages) was used to perform the negative binomial distribution method according to the two standard deviations where the absolute value of FC was greater than the median of FC. According to the hypergeometric distribution algorithm, the pathway enrichment analysis was carried out by querying GO biological process (BP), molecular function (MF) and cellular composition (CC) by DAVID (https://david.ncifcrf.gov). The same method was used in KEGG analysis. The cut-off value was p<0.05. Bioinformatics and Evolutionary Genomics (http://bioinformatics.psb.ugent.be/webtools/Venn) was used to select central or core genes.

### Clinical subjects

All lung tissues in this study were recruited from Tongji Hospital, Wuhan, China, between 2019 and 2021. All the patients involved were negative for SARS-CoV2 examinations. Thereinto, the lung specimens were collected from patients who underwent surgical resection for pulmonary lump in the department of thoracic surgery. All included participants were classified into two groups based on age, namely young COPD (≤ 50 years) and aged COPD (≥ 70 years). COPD was diagnosed according to the Global Initiative for Chronic Obstructive Lung Disease (GOLD) 2021 criteria. Patients with a post-bronchodilator forced expiratory volume in 1 s (FEV_1_)/forced vital capacity ratio of less than 70% were enrolled. Participants were excluded if they suffered from asthma, severe lung infections, or other obstructive lung diseases.

### Quantitative real-time polymerase chain reaction (qRT-PCR)

Total RNA of the lung tissue was extracted by RNAiso plus kit (TaKaRa) and reversely transcribed to cDNA using the cDNA RT-PCR Kit (Takara, Japan). The mRNA expression was assessed by real-time quantitative polymerase chain reaction (RT-qPCR) using TB GreenR Premix Ex TaqTM II (Takara, Japan) on BioRad CFX384 (Bio-Rad, CA, United States). The parameters were as follows: 40 cycles at 95° C for 10s, 59° C for 20s, and 72° C for 30s. Data were analyzed using the 2^−ΔΔCt^ method with β-actin as control. The primers were as follow: NKG7 (5’-CCAGAAGCCCTGAGCTTATCCC-3’ and 5’-AGTGAGCACCCAGGCTCAGGG-3’), CKLF (5’-TCGCTTCGCAGAACCTACTCA-3’ and 5’-TATTTTCGGCTGCACGTTATCC-3’), LRP4 (5’-GCCGCCAAGTCATTATCT-3’ and 5’-TCAGCACCTTCCTCTTACT-3’), MLF1 (5’-TCGTTTTTCCAATCTGTCCGC-3’ and 5’-GATACTGAGCAAGTCTCTTCC-3’), GDPD3 (5’-GCCAGTCGGGCCTAAACAG-3’ and 5’- GTCCTCCAGACGAACCATGC-3’), CXCL9 (5’- TGCAAGGAACCCCAGTAGTGA-3’ and 5’- GGTGGATAGTCCCTTGGTTGG-3’) and β-actin (5’- AGAAAATCTGGCACCACACCT-3’ and 5’- GATAGCACAGCCTGGATAGCA-3’).

### Statistical analysis

Data analysis was carried out by Rstudio (version 4.1.2) and GraphPad Prism 8 Software (GraphPad Software, San Diego, CA, United States). The microarray data were analyzed by different R packages. Results were expressed as mean ± SD or median (P25 quartile, P75 quartile). Statistical significance was determined using Student’s t-test for two groups. The correlations were analyzed by Pearson correlation. A two-sided p-value <0.05 was considered as statistically significant.

## Supplementary Material

Supplementary File 1

Supplementary File 2
